# Application and Comparison of Supervised Learning Strategies to Classify Polarity of Epithelial Cell Spheroids in 3D Culture

**DOI:** 10.3389/fgene.2020.00248

**Published:** 2020-03-27

**Authors:** Birga Soetje, Joachim Fuellekrug, Dieter Haffner, Wolfgang H. Ziegler

**Affiliations:** ^1^Department of Paediatric Kidney, Liver and Metabolic Diseases, Hannover Medical School, Hanover, Germany; ^2^Molecular Cell Biology Laboratory, Internal Medicine IV, University Hospital Heidelberg, Heidelberg, Germany

**Keywords:** image analysis, epithelial morphogenesis, 3D culture, polarity, spheroids, machine learning, deep learning, CNN

## Abstract

Three-dimensional culture systems that allow generation of monolayered epithelial cell spheroids are widely used to study epithelial function *in vitro*. Epithelial spheroid formation is applied to address cellular consequences of (mono)-genetic disorders, that is, ciliopathies, in toxicity testing, or to develop treatment options aimed to restore proper epithelial cell characteristics and function. With the potential of a high-throughput method, the main obstacle to efficient application of the spheroid formation assay so far is the laborious, time-consuming, and bias-prone analysis of spheroid images by individuals. Hundredths of multidimensional fluorescence images are blinded, rated by three persons, and subsequently, differences in ratings are compared and discussed. Here, we apply supervised learning and compare strategies based on machine learning versus deep learning. While deep learning approaches can directly process raw image data, machine learning requires transformed data of features extracted from fluorescence images. We verify the accuracy of both strategies on a validation data set, analyse an experimental data set, and observe that different strategies can be very accurate. Deep learning, however, is less sensitive to overfitting and experimental batch-to-batch variations, thus providing a rather powerful and easily adjustable classification tool.

## Introduction

Epithelia built from sheets of polarised cells line outer and inner surfaces of our body. The epithelial tissue found in many organs, such as kidneys, liver, lung, and the mammary gland, builds systems of ducts, tubules, and spherical cysts (O’Brien et al., 2002). These inner epithelia line cavities filled with fluids or gases, and organ function requires controlled barrier features of the epithelium. Epithelial barriers have an intrinsically asymmetric structure arising from different types of cell junctions. These junctions, as formed by specialised transmembrane receptor complexes, determine the physical interaction of cells and the barrier function of epithelial tissues (Bryant and Mostov, 2008).

Epithelial cells form (i) tight junctions (TJs), which constitute a barrier that physically separates the apical from the basolateral membrane compartment of epithelial cells and which also control paracellular transport across the epithelial sheet. In addition, (ii) adherens junctions (AJs) formed laterally, connect to the actin cytoskeleton and contribute to force transmission in the epithelial sheet (Wang and Margolis, 2007). Control of cell polarity is central to the establishment and maintenance of the epithelial barrier. The apical surface of cells is facing the lumen and acts as exchange interface for secretion and absorption processes. The junctional complexes (TJs and AJs) are required to establish and maintain polarisation and barrier function of epithelia. The basolateral surface is facing neighbouring cells in the epithelial sheet and extracellular matrix (ECM) at the outside (Roignot et al., 2013). Extracellular matrix is connected to the actin cytoskeleton of epithelial cells via (iii) integrin receptor–based cell–ECM adhesions. Epithelial lining of lumen is shaped by a fine spatiotemporal regulation of cell–cell and cell–ECM interactions (O’Brien et al., 2002).

Dysfunction of epithelia and luminal networks is observed in multiple tissues, for example, in the endothelium (Datta et al., 2011; Kovacic et al., 2012), in the intestine (Oshima and Miwa, 2016), or in the retinal epithelium of the eye (Hartnett, 2010). A further prominent example are ciliopathies, a rather heterogeneous group of rare inherited disorders, wherein defects manifest as different, mostly epithelial-derived disturbance of function such as retinal degeneration, cyst formation in kidney (and liver), and cerebral anomalies (Waters and Beales, 2011). To date, therapeutic management of ciliopathies is purely symptomatic and largely opinion-based (Ware et al., 2011; Ebner et al., 2015; Slaats et al., 2016). Thus, a better molecular understanding of the underlying pathomechanisms is crucial for the development of novel treatment options. Monogenetic disorders, like many ciliopathies (Hildebrandt et al., 2011), are proposed to cause cell autonomous defects, which should allow assessment of disease mechanisms and drug screening in cell culture–based assays.

In the past two decades, cell culture techniques enormously improved the understanding of cell and tissue function in a broad spectrum of research areas, which include developmental biology, tissue engineering, elucidation of disease mechanisms, toxicology, and drug discovery. At the same time, awareness of shortcomings in the ability of culture systems to emulate *in vivo* behaviour of cells was rising. Progress in cell culture methods enabled researchers to develop three-dimensional (3D) culture techniques, which allow cells to establish tissue-like cell–cell and cell–ECM interactions and define their 3D microenvironment and communication networks. These 3D culture conditions come much closer to a physiological *in vivo* situation than those commonly applied in 2D cell culture (Ravi et al., 2015). In 3D culture, epithelial cells form monolayered spheroids (also termed spheres, cysts, or acini) (Martin-Belmonte et al., 2008; Rodriguez-Fraticelli et al., 2012; Ivers et al., 2014; Fessenden et al., 2018), a miniaturised tissue that represents the simplest epithelial lumen–containing structure (Datta et al., 2011; Booij et al., 2019).

**FIGURE 1:**
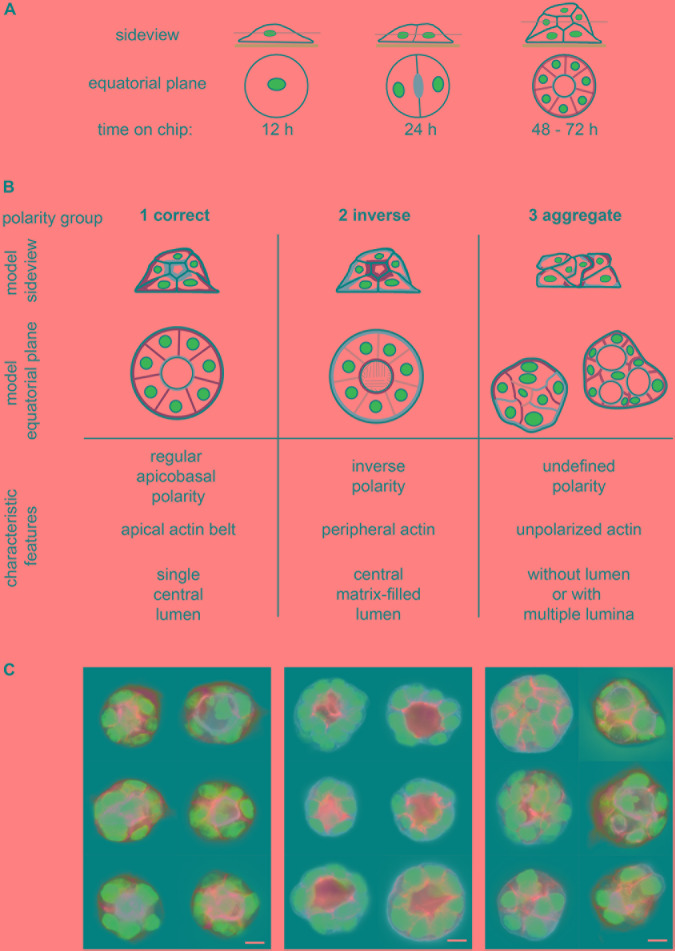
Assignment of epithelial cell spheroids to polarity groups. **(A)** Generation of spheroids; seeding of single MDCKII epithelial cells on micropattern (or in ECM gels) leads to spheroid formation within 3 days. Side view and equatorial plane section of spheroid formation on micropattern. **(B)** Definition of spheroid polarity groups; 1—correctly polarised, 2—inversely polarised, and 3—aggregates and multiple lumina. Upper panel: side view and equatorial plane of polarised spheroids showing (i) apical marker (e.g., gp135) or actin cytoskeleton in magenta, (ii) basolateral marker (e.g., gp58) in green, and (iii) nuclei in blue. Lower panel: characteristic features of polarity groups regarding polarity of membrane compartments, position of actin filament bundles, and 3D structure and lumen formation (for further description, see section “Results”). **(C)** Exemplary images of spheroid polarity groups showing apical marker (gp135, magenta), basolateral marker (gp58, green), and nuclei (blue). Bar: 10 μm.

In parallel to the progress in cell culture techniques, the requirement for adequate methods of analysis increased. To study cells cultured in 3D, image data acquisition requires adaptation to this situation. Especially in fluorescence microscopy techniques, the extension of images to a stack of z-planes in several colours led to huge image data sets that require appropriate processing tools. In addition, cell culture experiments, regardless of whether in 2D or 3D, increasingly require quantitative, statistically verified readouts. Thus, it is not feasible to draw conclusions from a drug treatment condition based on some 10 to 20 cells or spheroids. The demand for reproducible, spatially defined setups handling large numbers of cells (up to 100th) can be satisfied by using glass cover slips with micropatterned adhesion areas, so-called adhesion chips (e.g., from CYTOO S.A., Grenoble, France) (Rodriguez-Fraticelli et al., 2012). These chips provide adhesive micropatterns with a predefined shape, size, and density. In combination with a preselected ECM coating and adapted culture media, 3D-like culture conditions on adhesion chips allow generation of arrays of spheroids. Starting from separated single cells, epithelial cell spheroids form in homogenous conditions, cell type–dependent within 3 to 5 days (Figure 1A). Cell division is guided by defined ECM coating, spacing, and adhesion area, as well as Matrigel supplements of culture medium (Rodriguez-Fraticelli et al., 2012). These spheroids are accessible to high-resolution fluorescence microscopy for life-cell imaging and fixed cell approaches. When seeding cells into ECM gels, similar spheroids form, but it is not possible to define either the spacing of (groups of) cells or their z-positions, which considerably complicates image acquisition and statistical analysis of spheroid formation in gels.

To study epithelial cell function and morphogenesis, quantitative analysis of spheroid growth and polarity is most useful. Spheroid growth is employed in high-throughput approaches that test therapeutic treatment options, for example, in cyst development assays in polycystic kidney disease (Booij et al., 2017) or in cancer studies (Monjaret et al., 2016). More sophisticated analyses of spheroid growth and polarity are applied to assess consequences of genetic disorders (Hynes et al., 2014) and protein function (Deevi et al., 2014) and furthermore in mechanistic analyses of tissue morphogenesis and polarity establishment (Galvez-Santisteban et al., 2012; Petridou and Skourides, 2014).

Morphology of epithelial spheroids is classified based on characteristic features such as the spatial arrangement of cells and the force balance, observed by nuclei staining and actin filament bundles, respectively, and the distribution of protein markers for apical and basolateral surfaces to assess polarity. To date, analysis of spheroid polarity is performed by individuals who based on characteristic staining patterns classify spheroid images in different groups (Rodriguez-Fraticelli et al., 2012; Yonemura, 2014). First, this “manual” classification is influenced by personal bias or experience, for example, which distance of nuclei is enough to define a lumen, and second, it is rather time consuming.

To reduce restrictions and to allow rating for 100th of spheroids as required, for example, in treatment analysis of cells with different compounds, supervised learning is the strategy to automated classification. Supervised learning is achieved either by classical machine learning or more recently deep learning strategies. In the past decade, machine learning became important to the field of image analysis and is efficiently used for segmentation, feature extraction, and classification of image data (Sommer and Gerlich, 2013; Caicedo et al., 2017). In contrast to classical machine learning, which can be applied only to transformed image data, the benefit of deep learning is its ability to process raw image data (LeCun et al., 2015; Moen et al., 2019). This led to increasing importance of deep learning in biology and medicine supporting, first, bioinformatics analysis of protein function and prediction of pathway-related gene function (Le et al., 2018, 2019; Al-Ajlan and El Allali, 2019) and, second, diverse image-centred applications, such as segmentation, feature enhancement and recognition, and classification tasks, for optimised workflow in medical diagnosis (Esteva et al., 2017; Kermany et al., 2018; Alom et al., 2019; Tsochatzidis et al., 2019; Black et al., 2020), as well as reconstruction of superresolutional fluorescence images (Weigert et al., 2018; Belthangady and Royer, 2019) or cytometric high content analysis and phenotyping (Scheeder et al., 2018; Yao et al., 2019).

Here, we describe and compare two approaches of supervised learning based on machine learning and deep learning, respectively. The machine learning approach uses ImageJ/FIJI routines (Schindelin et al., 2012; Schindelin et al., 2015) and MATLAB (The MathWorks, Inc., Natick, MA, United States) for image preprocessing, feature extraction, and, finally, classifier training and classification. The deep learning–based approach using transfer learning of different pretrained CNNs is directly applied on image data. Performance and usability of the different supervised learning approaches are validated. While requiring considerably less time and personnel, these methods do not compromise the quality of spheroid rating and, in addition, provide tools to train and adapt the task of classification to variations in assay conditions, for example, newly arising spheroid phenotypes.

## Methods

### Biological Methods and Image Acquisition

#### Cell Culture and Spheroid Assay

Madin Darby canine kidney cells [MDCK II; #00062107, European Collection of Authenticated Cell Cultures, Salisbury, United Kingdom] are cultured in MEM (Sigma-Aldrich, Darmstadt, Germany) containing 5% FBS (Biowest, Nuaillé, France), 200 mM L-glutamine (Biochrom, Berlin, Germany), and 1% penicillin/streptomycin (Biochrom) and split every 3 to 4 days in ratios 1:10 to 1:15. Micropatterned chips (CYTOO S.A.) with disc-shaped micropatterns of 700 and 1600 μm^2^ are coated with collagen I (20 μg/mL; Sigma-Aldrich) and washed. There were 6 × 10^4^ cells per chip (four wells) seeded in MEM containing 2% FBS, 200 mM L-glutamine, and 1% penicillin/streptomycin. After 4 h, half of the medium is replaced by MEM containing, in addition, 5% Matrigel (Matrigel Basement Membrane Matrix, Corning, NY, United States). Within 3 days, single cells grow to spheroids of 12 to 24 cells. Spheroids are washed twice with 1 × phosphate-buffered saline (PBS), fixed with 4% paraformaldehyde in PBS for 15 min, permeabilised with 0.25% Triton-X-100 in PBS for 12 min, blocked for 1 h with 5% normal donkey serum (Merck Millipore, Darmstadt, Germany) in PBS, stained with antibodies and dyes (as detailed below), and mounted in Shandon Immu-Mount (Thermo Fisher Scientific, Waltham, MA, United States). Antibodies used were as follows: mouse-α-gp58 [β-subunit of Na^+^-K^+^-ATPase (Füllekrug et al., 2006)], mouse-α-gp135-Atto550-coupled [Podocalyxin; (Meder et al., 2005)], antibody production (Antibody Facility, TU-Braunschweig, Brunswick, Germany) purification and custom-labelling (Hypermol EK, Bielefeld, Germany); secondary antibodies: donkey anti–mouse IgG (H + L) secondary antibody, Alexa Fluor 488 conjugate (Invitrogen, Carlsbad, CA, United States); affinity staining: Alexa Fluor 660 phalloidin (Invitrogen), DAPI (0.25 μg/mL; Sigma-Aldrich). Details for application sample treatment are provided in Supplementary Material.

#### Imaging

Images were acquired on the Zeiss Axio Observer Z1 microscope, using the 63x Plan-Apochromat (NA 1.4) oil objective, the AxioCam MRm Rev.3 camera, and the software package AxioVision version 4.8.2 (all from Zeiss, Göttingen, Germany). In this setup, the optimal z-plane distance is 0.24 μm. Filter sets and related staining were as follows: (1) gp58-Alexa Fluor 488—filter set 38 HE, (2) gp135-Atto550—filter set 43 HE, (3) Phalloidin-Alexa Fluor 660—filter set 50, and (4) DAPI—filter set 49 (all filter sets from Zeiss). Summary images of each spheroid were acquired by fluorescence microscopy in four colours with 50 z-slices per spheroid and a typical ROI size of 256 × 256 pixels (or 512 × 512 pixels for large spheroids).

#### Computational Dependencies

ImageJ/FIJI macros for image preprocessing were developed using the ImageJ version 2.0.0-rc-59/1.51k (Schindelin et al., 2015) packaged in the FIJI distribution (Schindelin et al., 2012). MATLAB (The MathWorks, Inc.) scripts were implemented in version R2018b (version 9.5.0.944444). For training of the decision tree and usage of trained classifiers in MATLAB analysis with Classification Learner App, the “Statistics and Machine Learning Toolbox (version 11.4)” is required; for deep learning. the “Deep Learning Toolbox (version 12.0)” and Network Models (Deep Learning Toolbox Model for Inception-ResNet-v2 Network version 18.2.0, Deep Learning Toolbox Model for Inception-v3 Network version 18.2.0) were used.

## Results

### Spheroid Polarity and Manual Image Analysis

On micropatterned chips, single cells grow to spheroids of 12 to 24 cells within 3 days (Figure 1A). To stain spheroids for their polarity markers, we use monoclonal antisera specific for the apical marker protein gp135/podocalyxin (Meder et al., 2005) and the basolateral marker protein gp58 (β-subunit of Na^+^-K^+^-ATPase) (Füllekrug et al., 2006), as well as phalloidin and DAPI to stain F-actin and nuclei, respectively. Images of each spheroid are acquired by fluorescence microscopy in four colours with 50 z-slices per spheroid.

In our study, we define three groups of polarity: regular apicobasal polarity (group 1), inverse polarity (group 2), and aggregates (group 3) (Figures 1B,C). In group 1, the apicobasal polarity is characterised by a single central lumen with the apical surface of the cells facing the lumen and a pronounced apical actin belt marking the lumen. The basolateral surface is pointing to the outside. Group 2 shows inverse polarity with a basolateral, matrix-filled lumen inside, a peripheral actin ring, and the apical surface facing toward the outside of the spheroid. The aggregates of group 3 have an undefined polarity and display either no lumen (Figure 1C, group 3—left column) or multiple lumina (Figure 1C, group 3—right column). Aggregate or defective lumen formation results in further phenotypes as summarised together with related protein defects by Rodriguez-Fraticelli and Martin-Belmonte (2013).

In our laboratory, classification is performed in a blinded fashion by at least three persons grading each spheroid by the following criteria: lumen—3D position of nuclei, polarity—distribution of apical and basolateral markers, and force coupling—local enrichment of the actin network. Trained staff is able to classify approximately 80 to 100 spheroids per hour. Furthermore, all pictures have to be blinded, and inconsistent spheroid ratings require comparison and discussion by the group. Thus, “manual” classification is time consuming and bias-prone, depending on the training status and experience of the raters. Especially considering the development of a high-throughput assay system wherein 1000s of images are generated, a manual classification is not feasible.

### Outline of Automated Analysis

**FIGURE 2:**
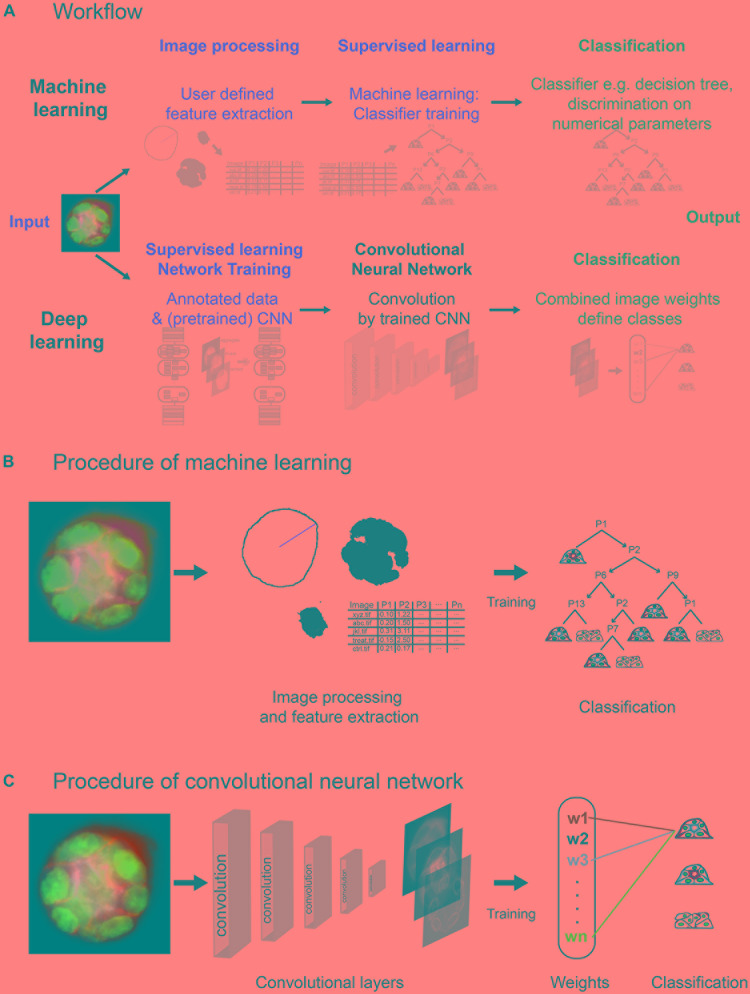
Layout of machine learning and deep learning strategies. **(A)** Workflow of approaches, showing computational steps as required for machine learning (upper panel) and deep learning (lower panel). Machine learning requires human input (red) in the steps of image processing and supervised learning to develop a classifier for spheroid classification. Deep learning requires human input in form of annotated data and a (pretrained) network within network training. Convolution of images and classification run within the network (CNN). **(B)** Machine learning comprises initial image processing and feature extraction steps. Equatorial plane images are processed to generate numerical parameters, which describe shape and/or structure of the spheroids, and radial distribution of fluorescence intensities. Upon training of a classifier, for example, classification tree, based on a subset of spheroid images, numerical parameters are used to determine the polarity of additional spheroids. **(C)** Convolutional neural networks reduce dimensionality applying different filters/kernels over a defined number of layers. This allows extraction of different local features such as edges in higher dimensional layers up to features of higher spatial distribution in deeper layers. Weights of these features are then combined in densely connected layers of the network to assign the classification label.

Spheroid polarity is radially symmetric at the equatorial plane. This feature is used to focus the analysis on a projection of an equatorial z-stack. We describe two types of supervised learning strategies, which are based on machine learning and deep learning, respectively (Figure 2A).

The machine learning–based analysis workflow requires processing of image data into inputs that are suitable for machine learning algorithms. Therefore, manually defined image processing and feature extraction steps are necessary to examine a number of descriptive shape parameters and the distributions of different fluorescent marker signals in the equatorial plane (Figure 2B and Supplementary Figures S1, S2). Using ImageJ/FIJI scripts, original images are processed, and shape parameters determined, feeding a set of MATLAB scripts with all information needed for further feature extraction. After polar transformation, cumulative signal intensities are plotted as a function of the radius, and positions and distances of the markers determined (Supplementary Figures S2A,B). Thereby, using the “Classification Learner App,” a set of 15 features for each spheroid is created (Supplementary Figure S2C) and used for training of a discriminative classifier. A classification tree is trained on a subset of spheroids, which were manually classified (at least 10–20 of each polarity group). To train an optimal classifier, features are analysed for their predictive power; different feature subsets are tested, and the most accurate subset is selected and applied for classifier training. An exemplary decision tree is visualised in Supplementary Figure S3. Thereafter, the trained classifier, for example, complex classification tree, can be employed to classify the bulk of spheroids (Figure 2B).

In contrast, the deep learning approach is based on transfer learning of open available CNNs trained on natural images, for example, GoogLeNet, Inception, ResNet, or, more optimal, a CNN trained for a similar purpose. Different deep CNN architectures were compared, for example, by Khan et al. (2019) and Shin et al. (2016). Spheroid’s equatorial plane projections are reduced to three colours, for example, basolateral surface in green, nuclei in blue, and actin or apical surface in red/magenta, and converted to 8-bit RGB images. Human input is limited to conversion of images to RGB, provision of annotated (classified) data, and choice of the CNN. Labelled images can be directly used to retrain one of these CNNs to replace weights and final classification layers for spheroid classification (Figure 2C). Within the CNN, depending on the structure of the network, the image is processed by convolution, applying different kernels/filters and thereby extracting features. Within the final classification layers of the network, a label, for example, dog—for natural images—or in our case, a correctly polarised spheroid of polarity group 1, is linked to the weights gained within the convolutional layers. The classification layers of a pretrained network are replaced by empty ones and retrained to labels such as polarity groups 1 to 3 (Supplementary Figure S4).

### Detailed Workflow for Machine Learning

#### Part 1—Image Processing and Feature Extraction in ImageJ/FIJI

The image analysis workflow is optimised for square image files featuring one spheroid only that is approximately centred within the xyz space and devoid of artefacts that could influence correct signal interpretation (Supplementary Figure S1A). These single-spheroid images can be directly acquired as ROIs during image acquisition or excised from z-stacks holding several spheroids in one image. To discriminate polarity, the following features are stained: (1) gp58 (or basolateral marker), (2) gp135 (or apical marker), (3) actin network, (4) nuclei. To extract descriptive shape parameters and projections of the equatorial plane, images are processed based on three functions (Figure 2B and Supplementary Figure S1): (i) <Midplane_MaxRadius> calculates the maximum radius, determines descriptive spheroid shape parameters, and extracts an equatorial plane as projection of central z-slices; (ii) <Actinbelt> calculates actin shape parameters on most intense actin signals displaying the contractile structures; and (iii) <CentreNuclei> extracts information on nuclear positions.

All shape parameters provided by the functions <Actinbelt>, <CentreNuclei>, and <Midplane_MaxRadius>, for further analysis in MATLAB, are summarised in Supplementary Figures S1C–E and listed in Supplementary Figure S2C. Equatorial plane images and results are saved.

#### Part 2—Analysis of Radial Intensity Profiles in Equatorial Plane Images (MATLAB)

In MATLAB, images of the equatorial plane and the position of the CoM from part 1 are used to analyse the spatial order of marker signals.

A stack of four-colour fluorescence images of spheroid equatorial planes is used for interpretation of marker positions. An angle-independent polar transformation (Supplementary Figure S2A) is performed by calculating the distance between each pixel (x_*i*_, y_*i*_) and the CoM of the spheroid and stored as a variable (Eq. 1).

(1) $$r\,a\,d\,i\,u\,s\,\left(x_{i},y_{i}\right)=\text{round}\,\sqrt{{\left(x_{i}-X_{\text{CoM}}\right)}^{2}+{\left(y_{i}-Y_{\text{CoM}}\right)}^{2}}$$

Subsequently, in each channel, intensity values of corresponding radii are integrated and divided by the number of pixels at each radius to calculate the mean intensity value per pixel. These mean intensity values are further normalised by the sum of all intensity values for that channel. A cumulative intensity curve is calculated by adding up all intensities up to each radius, and the relative radial position is calculated by dividing through the maximum radius. The localisation of polarity markers can be visualised by plotting cumulated channel intensities versus the relative radius (Supplementary Figure S2B). To determine representative positional information from these cumulative intensity curves, the radius position at 60% of the total intensity value is retrieved for each channel/marker. The radial distance at an intensity of 60% is used as approximated centre of the linear slope within cumulative marker curves (Supplementary Figure S2B). The initial increase of the nuclear signal is approximated by use of the cumulative intensity at 30% of spheroid radius and calculation of a linear slope. The initial slope of the nuclear signal is used to report the presence of nuclei within the expected luminal area (Supplementary Figure S2B). To increase predictive power of signal’s relative radius positions, differences of the polarity markers gp58 to gp135 and actin to gp135 are used. The relative position of gp58, the basolateral marker, to gp135, the apical marker, is a key feature of apicobasal polarity. A positive value Δgp58-gp135 indicates regular apicobasal polarity, whereas a negative value suggests inverse polarity. The consistency of the polarity assignment is verified using Δactin-gp135. Distinctly polarised spheroids show high colocalisation, that is, no or very small difference of actin and gp135, the apical marker, whereas high differences indicate a tendency of undefined polarity.

Altogether, 15 numerical parameters/features are generated from the spheroid images to allow usage of supervised machine learning algorithms for spheroid polarity classification. The detailed list of parameters is given in Supplementary Figure S2C. The most important features with the highest predictive power are as follows: (1) difference of polarity markers (gp58-gp135), (2) difference of actin to gp135, (8) circularity of nuclei, (9) average initial slope of nuclear signal, (10) relative actin area, (11) actin particle count, (14) distance CoM nuclei to CoM spheroid.

#### Part 3—Classifier Training and Classification

The MATLAB “Classification Learner App” was applied to an exemplary data set (*n* = 120 spheroids) selecting the complex decision tree algorithm with 20-fold cross validation in order to avoid overfitting. Other possible algorithms for discriminating approaches are, for example, support vector machines, nearest neighbour classifiers, or random forest ensembles (Sommer and Gerlich, 2013). Estimation of the predictor importance can be used to define the optimal subset of predictors and additional usage of the hyperparameter optimisation tool within the “Classification Learner App” will help to choose the best parameters for split criteria. In our example, using a subset of 12 from 15 parameters, more consistent and accurate results are obtained. Reduction of parameters leads to decreased dimensionality of the classification task and by this to less complex decisions. The decision tree for the exemplary analysis is shown in Supplementary Figure S3.

Revealing limitations of a single complex decision tree, application of this classifier to an exemplary data set of 154 spheroids resulted in a discrepancy of 18% as compared to the manual assignment, mainly affecting polarity groups 1 and 3. To resolve this discrepancy, a second classification step can be added, using another subset of features, for example, with best discriminative power for polarity groups 1 and 3, thus providing a rationale for the necessity and the outcome of reclassification. Alternatively, an ensemble of classifiers can be used. Finally, a table containing all pieces of information—image names, classification parameters, classification results, and reclassification decision—is generated.

### Detailed Workflow for Deep Learning

#### Part 1—Image Processing

In contrast to machine learning algorithms, deep neural networks are able to use images as direct input, performing feature extraction and classification within the network. For image-based decisions, the common architecture is a CNN (Figure 2C). In brief, usage of filters/kernels allows feature extraction from low-level features with reduced spatial dimensions such as edges within the first few layers of the network up to specific high-level features of higher spatial distribution such as overall shape in the deeper layers. Pooling layers are used to highlight the most dominant features and reduce required computational power. Within these convolution steps, general features of spheroids are recognised. Specific structure and connected layers depend on the chosen network architecture, which is usually highly complex. For example, the Inception v3 architecture contains 42 layers of convolution, pooling, inception modules, grid size reduction, and auxiliary classifiers, reducing images from a dimensionality of 299 × 299 × 3 to 8 × 8 × 2048 with final pooling to 1 × 1 × 2048 and classification to 1 × 1 × labels (Szegedy et al., 2015). The defined input size of the networks chosen for this analysis, Inception v3 and InceptionResNet v2, requires input of an RGB or three-channel image. Therefore, original images were converted to 8-bit RGB input images based on a z-projection of the equatorial plane. The four-colour fluorescence information was reduced to gp58 (basolateral marker, green), nuclei (DAPI, blue), and either gp135 (apical marker, red) or actin (apical ring, magenta) because of their high correspondence. Images with more than one spheroid were split, previously.

#### Part 2—Network Training, Transfer Learning

Training of an efficient and reliable convolutional network from scratch needs a very high number of labelled training image data sets. To bypass this obstacle, an efficient and widely used method is to use “transfer learning” of a network, which should be trained optimally on similar image data or if not available on natural images, for example, GoogLeNet, Inception, or ResNet (Szegedy et al., 2015; Gupta et al., 2019; Moen et al., 2019; Tang et al., 2019), and to retrain the final layers necessary for prediction and classification (Supplementary Figure S4). Most openly available CNNs are validated on the ILSVRC 2012 classification challenge validation set and can be directly compared. Final layers of the chosen CNNs (Inception v3 and InceptionResNet v2) are replaced by empty ones, and the network is retrained iteratively to define the weights and biases of the neurons, concluding from extracted features to a single class label (Figure 2C). Training was performed in 15 epochs (full training cycle with entire training data) with batch size of *n* = 80 spheroids and a learning rate of 3 × 10^–3^ on a data set of *n* = 698 spheroids inhomogeneously distributed in four polarity groups. The number of epochs, the learning rate, and the batch size per iteration can be varied. The SGDM (Stochastic Gradient Descent With Momentum) method was chosen as optimiser. Within the training, internal validation is integrated to optimise accuracy and to minimise the “loss function” serving as discrepancy indicator for correct classification.

#### Part 3—Validation and Classification

Using the retrained network, classes of a validation data set can be predicted, and the accuracy of this decision validated. Validation is an important step, to recognise if, for example, the number of labels/classes is sufficient or if heterogeneous groups should be separated in different labels. For example, the group of aggregates (Figure 1A) was separated in (i) aggregates without lumen and with undefined polarity and in (ii) aggregates with multiple lumina. Networks can be retrained again, to accommodate new class labels or other changes.

### Validation of the Classification Method Using Cohen’s κ

For the validation of mostly diagnostic categorisation systems, comparison of interrater reliability is a widely used reference tool to match results across two or more test systems. A rater can either be an individual or the automated classification algorithm. As initial estimate, the percent agreement (accuracy) is an easily accessible, directly interpretable value, calculated by dividing the count of alike-classified spheroids by the total number of spheroids. Additionally, recall (sensitivity) and precision (positive predictive value) for group 1 classification were calculated. The combination of percentage agreement and cross-correlation table reveals limitations of the assignment of chosen classes. A key limitation in use of the percentage agreement is the possibility of raters to guess the group, which is not taken into account (McHugh, 2012). Similar to a correlation coefficient, Jacob Cohen (1960) developed a nominal reliability coefficient κ, which scores the grade of agreement and provides an error and confidence interval to describe the coefficient. Values of κ range from −1 to + 1. Whereas values below 0 represent disagreement; 0 signifies random agreement, and values greater than 0 denote increasing levels of agreement. An interpretation of κ values is suggested as follows: 0.01 to 0.20 as *none* to *slight*, 0.21 to 0.40 as *fair*, 0.41 to 0.60 as *moderate*, 0.61 to 0.80 as *substantial*, and 0.81 to 1.00 as *almost perfect* agreement (McHugh, 2012).

Statistical calculation of the Cohen’s κ value by the MATLAB script Cohen’s κ (Cardillo, 2007) was performed after computing a cross-correlation table for two different raters and three classification groups based on comparison of the results of classification for every spheroid. In addition, the correlation was verified using the web services “ReCal2” for two raters, that is, two manual classifications or manual versus automated classification, and “ReCal3” for three or more raters, that is, in the case of manual classification by more than two individuals (Freelon, 2010, 2013).

### Method Validation and Application Example

Semiautomated spheroid classification is implemented as a combination of self-written ImageJ macros and MATLAB scripts and by using MATLAB “Statistics and Machine Learning Toolbox,” “Deep Learning Toolbox,” and Network Models (Inception-ResNet-v2 Network, Inception-v3 Network).

For the machine learning–based approach, altogether *n* = 120 spheroids or approximately 20 to 40 spheroids of each polarity group: (1) regular apicobasal polarity, (2) inversed polarity, and (3) aggregates or multiple lumina (Figure 1B), were used within the “Classification Learner App.” Initial accuracy of different algorithms, for example, decision trees, support vector machines, or nearest neighbour classifiers, was tested, and we decided for complex decision trees because of a high accuracy and a high degree of user acceptance caused by interpretability of decisions. Furthermore, estimation of the predictor importance was used to define the optimal subset of predictors. As described, for our data, the complex decision tree algorithm with 20-fold cross validation and a subset of 12 of 15 parameters (parameters 1, 2, and 6–15; Supplementary Figure S2C) achieved the best score. As a result of the classifier training, the complex decision tree classifier is generated for subsequent classification (Supplementary Figure S3).

**FIGURE 3:**
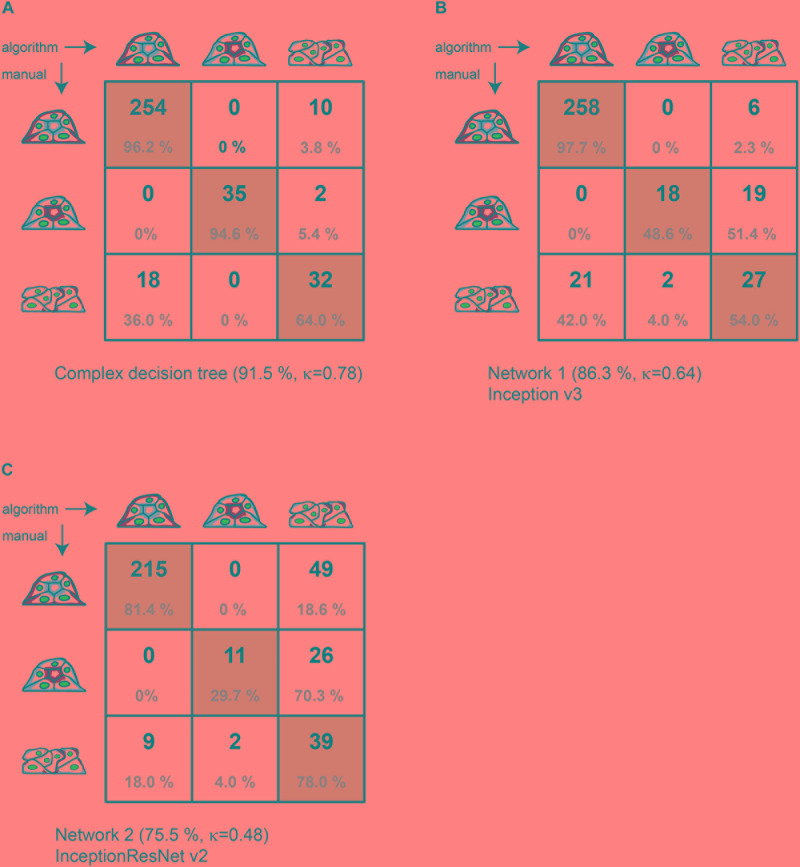
Comparison of manual to automated classification based on a validation data set. Cross-correlation table of “agreed” manual classification versus classification predicted by **(A)** the complex decision tree classifier, **(B)** the Inception v3-based CNN, or **(C)** the InceptionResNet v2-based CNN. Cells with identical classification are highlighted in green. Summary lines indicate overall percentage of agreement and Cohen’s k of interrater agreement.

To probe reliability of polarity classification, a set of randomly selected MDCK II spheroids (*n* = 351) was analysed using the (i) manual classification, (ii) a complex decision tree classifier together with resorting by a set of decision trees, and (iii + iv) CNNs based on Inception v3 and InceptionResNet v2. Both CNNs were trained on a bigger data set of *n* = 531 spheroids. Based on manual classification by three raters, the distribution of spheroids was 75.2% (*n* = 264) of group 1, 10.5% (*n* = 37) of group 2, and 14.2% (*n* = 50) of group 3 (Figure 3). The inverse spheroids of group 2 represent a rare event and lead to uneven group distribution, revealing limitations of every supervised learning strategy as these require even distributions of all groups for optimal function.

Figure 3 provides the detailed comparison of group assignments by all three methods in respective cross-correlation tables. With 321 of 351 spheroids classified identically, the “agreed” manual classification (three raters with discussion of results) and the machine learning–based classification (with reclassification) achieve 91.5% of agreement. This value is substantially higher than the consent between two manual raters before discussion (80.2%). The value for Cohen’s interrater agreement is κ = 0.78 [SE κ = 0.038; CI (α = 0.05) = 0.705 to 0.855], revealing substantial agreement of both classification methods (recall = 0.962; precision = 0.934) (Figure 3A). With a percentage agreement of 86.3%, the manual classification and the Inception v3–based CNN also show substantial agreement of κ = 0.64 [SE κ = 0.049; CI (α = 0.05) = 0.540 to 0.731] (recall = 0.977; precision = 0.925) (Figure 3B). Classification of the CNN based on InceptionResNet v2 resulted in moderate agreement of 75.5% with a κ = 0.48 [SE κ = 0.049; CI (α = 0.05) = 0.383 to 0.574] (recall = 0.960; precision = 0.814) (Figure 3C).

### Application to an Experimental Data Set

**FIGURE 4:**
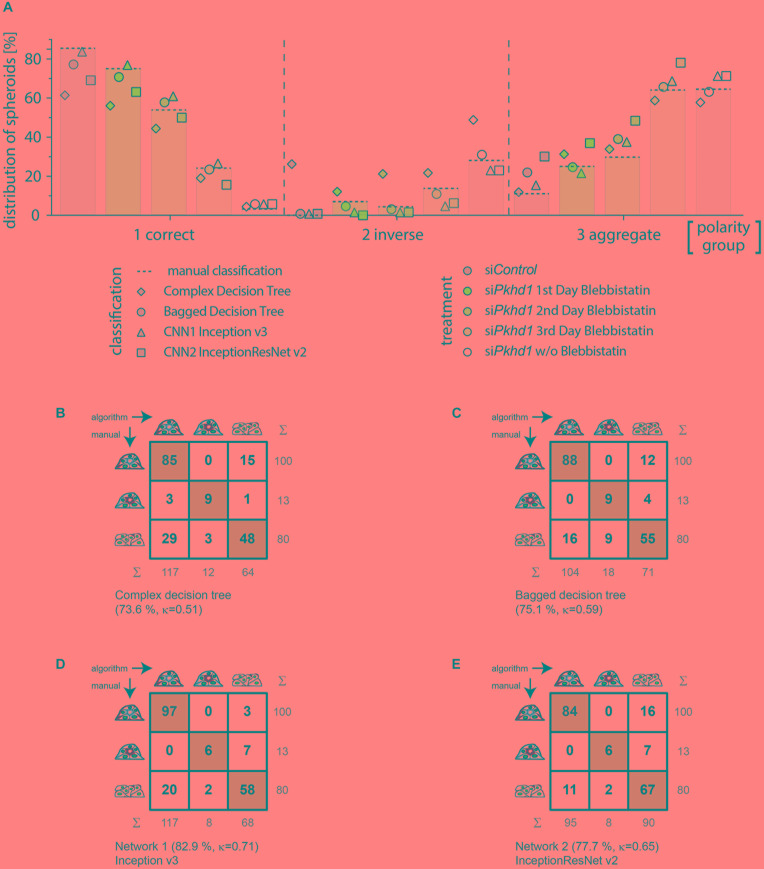
Classification of an experimental data set comparing cross-correlation tables of manual versus automated group assignment. **(A)** Classification results on experimental data determined manually (bars and dotted line), by complex decision tree classifier (diamond), bagged decision tree classifier (circle), Inception v3-based CNN (triangle), and InceptionResNet v2-based CNN (square). MDCKII *Pkhd1* knockdown cells (si*Pkhd1*) and controls (si*Control*) were seeded individually to allow spheroid morphogenesis for 3 days and treated with blebbistatin either on day 1, 2, or 3 or kept untreated during this period of time. Colours indicate treatment groups. Data were generated in four independent experiments (*n* = 698 spheroids; 122–182 spheroids per condition). Cross-correlation table of “agreed” manual classification versus classification predicted by **(B)** complex decision tree classifier, **(C)** bagged decision tree, **(D)** Inception v3-based CNN, or **(E)** InceptionResNet v2-based CNN. Cells with identical classification are highlighted in green. Summary lines indicate percentage of agreement and Cohen’s k interrater agreement.

To compare accuracy and reliability of both types of supervised learning strategies directly on more diverse data, an experimental data set was selected. In brief, consequences of siRNA-mediated knockdown of *Pkhd1* and subsequent treatment, on epithelial morphogenesis, were analysed. MDCKII cells were treated with si*Control* or si*Pkhd1* and single cells seeded onto disc-shaped micropatterns to study their capacity of spheroid formation. Si*Pkhd1*-treated cells were additionally treated with blebbistatin, a myosin II inhibitor, either on day 1, 2, or 3 of spheroid growth. Figure 4 shows results of manual classification compared to machine learning–based classification by the complex decision tree with resorting (as described above), a freshly trained classifier of bagged decision trees and classification by the two CNNs based on Inception v3 and InceptionResNet v2. The experimental data set consisted of *n* = 698 images from four independent experiments. Images of experiments 1 to 3 (*n* = 531 images) were used to train the bagged decision trees and to retrain the two CNNs. The complex decision tree was trained on a different data set, acquired months before the experimental data set. Distribution of spheroids in the experimental data set was as follows: 153 spheroids of group 1, 121 spheroids of group 2, and 257 spheroids of group 3 (226 aggregates and 31 multilumen). Experiment 4 (*n* = 193 images) was used to validate and compare performance of the four different methods. The distribution of spheroids in the validation data of experiment 4 was as follows: 51.8% (*n* = 100) of group 1, 6.7% (*n* = 13) of group 2, and 42.5% of group 3 (*n* = 80 with 60 aggregates and 20 spheroids with multiple lumina).

Figure 4A illustrates the classification results of the entire experimental data set (experiments 1–4), wherein bars with dotted line indicate manual classification. Symbols represent classification by the different supervised learning algorithms, and colours specify treatment of cells. In spheroids of group 1, classification of the bagged decision tree (circle) and the Inception v3–based network 1 (triangle) show the highest agreement. Classification of group 2 spheroids is well-represented by bagged decision tree (circle) followed by the Inception v3–based network 1 (triangle) and InceptionResNet v2 based network 2 (square). Group 3 spheroids classification seems to be similarly accurate in complex decision tree (diamond) and bagged decision tree (circle), followed by the Inception v3–based network 1 (triangle). Analysing the sum of absolute percentage differences, that is, disagreement from manual classification as ground truth for all three polarity groups and treatments, results are very high for the complex classification tree classifier (359%). The bagged classification tree shows the lowest difference (31%), and both CNN-based classifications differ to a somewhat higher amount with 63% and 71% for Inception v3 and InceptionResNet v2, respectively.

In contrast to its performance on the validation data set, complex decision tree classification of the experimental data set resulted in moderate agreement of 73.6% (κ = 0.51) (recall = 0.795; precision = 0.644). The cross-correlation table (Figure 4B) reveals clear limitations in the discrimination of groups 1 and 3, resulting in overestimation of correctly polarised spheroids and underestimation of aggregates. The distribution was 60.6% (*n* = 117) of group 1, 6.2% (*n* = 12) of group 2, and 33.2% (*n* = 64) of group 3. The observed lack of accuracy as compared to classification of the validation data set probably arises from the numerical dependence of decision tree-based classification. Separators used within the classifiers tend to be overfitting for training data sets. Thus, data sets acquired weeks or months later often necessitate adaption, which means complete retraining of the decision trees. Consistently, the ensemble of bagged decision trees, freshly trained on a data set more similar to the experimental data set (experiments 1–3), resulted in a better percentage agreement of 75.1% and a better Cohen’s κ value of 0.59 (recall = 0.886; precision = 0.946) (Figure 4C). The bagged tree classification determined a distribution of 53.9% (*n* = 104) of group 1, 9.3% (*n* = 18) of group 2, and 36.8% (*n* = 71) of group 3, with very similar numbers of spheroids divergently assigned to groups 1 and 3. Machine learning–based classifiers are prone to loose accuracy due to normal variation of cell biological assay conditions.

In comparison, the deep learning approaches using Inception v3 (Iv3) (Figure 4D) and InceptionResNet v2 (IRNv2) (Figure 4E) networks, which were retrained by transfer learning, resulted in percentage agreements of 82.9% for Iv3 and 77.7% for IRNv2, or Cohen’s κ of 0.71 and 0.65 respectively, showing substantial agreement levels as determined also on the validation data set (recall = 0.970 and 0.840, precision = 0.829 and 0.884). Group distributions for Iv3 were 60.6% (*n* = 117) of group 1, 4.2% (*n* = 8) of group 2, and 35.2% of group 3 (*n* = 68; 61 aggregates and 7 multilumen), and, for IRNv2 49.2% (*n* = 95) of group 1, 4.2% (*n* = 8) of group 2, and 46.6% of group 3 (*n* = 90; 61 aggregates and 29 multilumen). While the total number of discrepancies is lower for the Iv3-based CNN, the cross-correlation table (Figure 4D) reveals unbalanced divergent classification of groups 1 and 3 by this network. The IRNv2-based network, in contrast, shows a slightly decreased accuracy but a balanced number of groups 1 and 3 divergent classifications. A high and unbalanced number of divergent assignments to groups 1 and 3 can influence the outcome of an experiment revealing the demand for high accuracy and balanced distribution of disputed decisions.

## Discussion

Epithelial spheroid formation in 3D cell culture constitutes a recognised and widely used *in vitro* model addressing the capability of cells to perform correct epithelial morphogenesis. The model was used for multiple applications and in cells of different tissue origin (Debnath and Brugge, 2005; Giles et al., 2014; Okuyama et al., 2016; Booij et al., 2017). To date, all approaches share the common restriction of limited cell/spheroid numbers and the laborious step of a manual classification of apicobasal polarity. By establishing a (semi)automated method, the time-consuming and bias-prone aspect of epithelial polarity classification can be reduced to less than one-fifth of its duration or even lower without loss in quality of the experimental outcome (Figure 4A).

### Performance of Supervised Learning in Relation to Manual Classification

First, manual classification needs generation of blinded image data, because the rater should not be able to deduce experimental conditions from image names or other sources of information. In addition, for a skilled/trained rater, classification of 50 spheroid image files (four-colour z-stacks, 50 planes) requires 30 min or more of focussed attention and, moreover, has to be performed by at least two, better three analysts. After initial classification, the results of all analysts are compared and, in case of disagreement/inconsistencies, require discussion in the group to find an agreement. In contrast, the duration of semiautomated analysis by machine learning for 50 spheroid image files is approximately 20 min from loading of the images to the results table [duration estimated using a desktop computer; Intel Xeon X5650 at 2.67 GHz (two processors), 16 GB RAM]. Even if times for classifier training (and reclassification) as needed for semiautomated analysis are taken into account, a reduction of analysis time by 80% is realistic. Deep learning approaches using pretrained CNNs are even faster. The time-consuming step of network training took approximately 6 h for the most complex network based on InceptionResNet v2 on one CPU, parallel computing on GPU, or multiple CPUs can speed up the training even for larger training data sets. The duration of prediction only takes up to a few minutes. In contrast, net analysis times for manual classification are high, directly proportional to the number of spheroids and cannot be reduced significantly by (further) training of the analysts. This constitutes a relevant limitation of all studies using manual classification.

Second, comparison of percentage agreement and interrater reliability is an essential criterion for the validation of subjectively categorised data and is widely used in medical sciences to validate diagnostic categorisation systems (Freelon, 2010). In the exemplary analysis of the validation data set, an agreement of 80.2% for the manual classification of *n* = 351 spheroids by at least two raters was observed, revealing that in approximately 20% of the data raters initially disagree in their classifications. The level of disagreement highlights that the scoring process needs training and discussion of results. As aforementioned, the Cohen’s coefficient κ is a parameter better suited to compare nominal reliability.

In our example of the validation data set, the interrater agreement of manual classification reached κ = 0.60, which represents a moderate agreement. Comparison of the “agreed” manual classification and the semiautomated classification revealed substantial consent of 91.5% with κ = 0.78. Thus, semiautomated classification can be considered an adequate and time-saving match to manual classification without compromising the quality of analysis. However, applying the same machine learning–based classifier on a similar but unrelated data, the experimental data set, the value of percentage agreement decreased to 73.6% and κ = 0.51 for the complex decision tree classifier. Furthermore, the bagged decision tree classifier, which was trained on a related data set, slightly increased the percentage agreement to 75.1% with κ = 0.59. Thus, machine learning–based approaches are able to perform reliable classifications as demonstrated for the validation data set and the freshly trained tree classifier but are limited by their dependence on numerical separators. The batch-to-batch variability of biological experiments is an obstacle, leading to decreasing accuracy on new data sets and requiring much effort to ensure accuracy and sensitivity of the classification algorithm. Optimal results are only achieved on highly similar data sets and furthermore demand rigorous control and continuous validation.

The outcome of deep learning classifiers with respect to accuracy and reliability is consistently less sensitive to batch-to-batch variations in biological data sets. The values for the Iv3-based CNN were high in both data sets with a substantial agreement 86.3% and κ = 0.64 for the unrelated validation data set compared to 82.9% and κ = 0.71 for the related experimental data set. Validation of the IRNv2-based classification resulted in moderate agreement levels for the unrelated validation data set with 75.5% and κ = 0.48 and substantial agreement of 77.7% and κ = 0.65 for the related experimental data set. Testing different network structures with differing layer numbers is important because deeper networks do not necessarily result in a higher accuracy.

### Comparison of Supervised Learning Strategies

Considering the small number of images for network retraining, wherein the smallest group of multilumen aggregates contained only 31 images, and the other groups between 121 and 226 images, the immediate performance of the network indicates a high potential to replace manual classification in a reliable manner. Small image numbers can be boosted by image augmentation, for example, rotation, mirroring, shifting, and resizing of images. By this means also overfitting of the network can be overcome, resulting in more accurate and powerful networks for spheroid classification and low susceptibility for batch-to-batch variations. Data augmentation by random rotation (45–315°) and scaling (0.4–1.2) of the same training data to n = 600 spheroids per label increased accuracy of the IRNv2-based CNN to 81.8% (κ = 0.56) for the validation data set from Figure 3 and to 85.0% (κ = 0.71) for the experimental data set from Figure 4. An increasing number of training data sets from different situations will enable further optimised classification results by the network. Additionally, specific group definitions are very important using CNN-based image classification. For example, subdivision of group 3 into aggregates and spheroids with multiple lumina (Figures 1B,C) led to an improvement of the initial validation accuracies by 5% to 10%.

## Conclusion

Assessment of epithelial function is central for the understanding of disease mechanisms and development of treatment options for many epithelia-derived states of disease such as ciliopathies. Classification of epithelial spheroid polarity in 3D culture is a well-established method that allows assessment of cell function in lumen formation and establishment of apicobasal polarity as required for the analysis of disease states. With the introduction of periodically structured adhesion chips to 3D culture, tightly controlled assay conditions can be combined with high-resolution automated imaging. The system is well-suited for adaptation to high content and high-throughput analyses. Functional studies on a couple of 100 of epithelial spheroids with the capacity to screen for subtle effects otherwise attributed to normal biological variation become realistic.

Thus, classification of spheroid polarity requires an adequate analysis technique allowing reliable group assignment of high numbers of multidimensional images. In a machine learning–based approach, we developed a combination of ImageJ/FIJI and MATLAB scripts, and in a deep learning–based approach, we tested retraining of two CNNs for spheroid classification. Both methods were shown (i) to at least match the current standard of an “agreed” classification by three raters and (ii) to reduce the estimated analysis time by at least fivefold (or even more for large data sets and deep learning approaches). In addition, the machine learning workflow generates (iii) a comprehensive results table with quantitative features of all spheroids analysed, whereas the CNNs provide (iv) a probability of correct group decisions. This output helps in understanding of the decision process.

We are convinced that our implementation of ImageJ/FIJI and MATLAB scripts using supervised learning is well-suited to match the requirements of an automated analysis of epithelial spheroid polarity. Bearing in mind, however, that the scripting for adaption to other image characteristics, for example, staining or other morphological properties, in feature extraction of the machine learning approach demands a trained and skilled image analyst, the deep learning approach is the more useful and readily available access to a supervised learning strategy in spheroid classification. Based on equatorial plane images as RGB, deep learning based approaches negate the need of intense image processing and feature extraction. Even in training and usage, the CNNs with MATLAB is less complex and results in reliable classifications outperforming the machine learning approach on unrelated data sets. Optimisation for higher performance can be achieved by increased training data numbers and/or data augmentation in the training period.

### Outlook

The workflow for classification of spheroid polarity was developed on wide-field fluorescence microscopy images for reasons of time-economic imaging and access to instruments that enable high-throughput analysis. Other imaging techniques with higher resolution in z-direction such as confocal microscopy, spinning disc, or light sheet fluorescence microscopy can improve contrast and therefore are expected to work alike or better, especially when using other marker proteins such as receptors or ion channels.

## Data Availability Statement

Project name: Spheroid polarity.

Operating system(s): Platform independent. ImageJ version 2.0.0-rc-59/1.51k including the FIJI image processing package, MATLAB (R2018b). MATLAB Statistics and Machine Learning Toolbox, Deep Learning Toolbox (v. 12.0), Network Models (Deep Learning Toolbox Model for Inception-ResNet-v2 Network v. 18.2.0, Deep Learning Toolbox Model for Inception-v3 Network v. 18.2.0).

Programming language: ImageJ macro language, MATLAB scripting language (R2018b).

Other requirements: *cell2string* function from MATLAB file exchange (Huang, 2013).

Licence: GPLv3.

Data availability statements: Code is provided on GitHub and in the Cloud. Current ImageJ macros and MATLAB scripts are deposited on GitHub, https://github.com/WolfgangZiegler/epithelial_spheroids. Exemplary images files and a fully annotated data set are available at https://owncloud-shib.gwdg.de/index.php/s/atBSGxKSFTemHuN.

## Author Contributions

BS designed and implemented the ImageJ macros and MATLAB scripts. WZ and DH contributed to design and structure of the automated classification and data analysis. BS performed biological experiments and BS and WZ analysed data. BS and WZ wrote the main manuscript and prepared the figures. JF provided biological tools for discrimination of polarity and contributed to the manuscript text. All authors reviewed the manuscript.

## Conflict of Interest

The authors declare that the research was conducted in the absence of any commercial or financial relationships that could be construed as a potential conflict of interest.

## Abbreviations

2D: two dimensional
3D: three dimensional
AJ: adherens junction
CI: confidence interval
CNN: convolutional neural network
CoM: centre of mass
DAPI: 4′,6-diamidin-2-phenylindol
ECM: extracellular matrix
F-actin: filamentous actin
FBS: foetal bovine serum
gp58: glycoprotein 58 (β-subunit of Na^+^-K^+^-ATPase)
gp135: glycoprotein 135 (podocalyxin)
Iv3: Inception v3 network architecture
IRNv2: InceptionResNet v2 network architecture
MDCK: Madin Darby canine kidney
MEM: minimal essential medium
NA: numerical aperture
PBS: phosphate-buffered saline
RGB: Red-Green-Blue colour code
ROI: region of interest
SE: standard error
TJ: tight junction
ZO-1: zona occludens-1 protein.

## References

[B1] Al-Ajlan A. El Allali A. (2019). CNN-MGP: convolutional neural networks for metagenomics gene prediction. *Interdiscip. Sci. Comput. Life Sci.* 114 628–635. 10.1007/s12539-018-0313-4 30588558 PMC6841655

[B2] Alom M. Z. Yakopcic C. Nasrin M. S. Taha T. M. Asari V. K. (2019). Breast cancer classification from histopathological images with inception recurrent residual convolutional neural network. *J. Digital Imaging* 324 605–617. 10.1007/s10278-019-00182-7 30756265 PMC6646497

[B3] Belthangady C. Royer L. A. (2019). Applications, promises, and pitfalls of deep learning for fluorescence image reconstruction. *Nat. Methods* 16 1215–1225. 10.1038/s41592-019-0458-z 31285623

[B4] Black K. M. Law H. Aldouhki A. Deng J. Ghani K. R. (2020). Deep learning computer vision algorithm for detecting kidney stone composition. *BJU Int.* [Epub ahead of print]. 10.1111/bju.15035 32045113

[B5] Booij T. H. Bange H. Leonhard W. N. Yan K. Fokkelman M. Kunnen S. J. (2017). High-throughput phenotypic screening of kinase inhibitors to identify drug targets for polycystic kidney disease. *SLAS Discov.* 228 974–984. 10.1177/2472555217716056 28644734 PMC5574491

[B6] Booij T. H. Price L. S. Danen E. H. J. (2019). 3D cell-based assays for drug screens: challenges in imaging, image analysis, and high-content analysis. *SLAS Discov.* 246 615–627. 10.1177/2472555219830087 30817892 PMC6589915

[B7] Bryant D. M. Mostov K. E. (2008). From cells to organs: building polarized tissue. *Nat. Rev. Mol. Cell Biol.* 911 887–901. 10.1038/nrm2523 18946477 PMC2921794

[B8] Caicedo J. C. Cooper S. Heigwer F. Warchal S. Qiu P. Molnar C. (2017). Data-analysis strategies for image-based cell profiling. *Nat. Methods* 14 849–863. 28858338 10.1038/nmeth.4397 PMC6871000

[B9] Cardillo G. (2007). *Cohen’s Kappa: Compute the Cohen’s Kappa Ratio on a 2 × 2 Matrix*. Available online at: http://www.mathworks.com/matlabcentral/fileexchange/15365 (accessed Februrary 24, 2017).

[B10] Cohen J. (1960). A coefficient of agreement for nominal scales. *Educ. Psychol. Meas.* 201 37–46. 10.1177/001316446002000104

[B11] Datta A. Bryant D. M. Mostov K. E. (2011). Molecular regulation of lumen morphogenesis. *Curr. Biol.* 213 R126–R136. 10.1016/j.cub.2010.12.003 PMC3771703 21300279

[B12] Debnath J. Brugge J. S. (2005). Modelling glandular epithelial cancers in three-dimensional cultures. *Nat. Rev. Cancer* 59 675–688. 10.1038/nrc1695 16148884

[B13] Deevi R. K. Cox O. T. O’Connor R. (2014). Essential function for PDLIM2 in cell polarization in three-dimensional cultures by feedback regulation of the beta1-integrin-RhoA signaling axis. *Neoplasia* 165 422–431. 10.1016/j.neo.2014.04.006 24863845 PMC4198691

[B14] Ebner K. Feldkoetter M. Ariceta G. Bergmann C. Buettner R. Doyon A. (2015). Rationale, design and objectives of ARegPKD, a European ARPKD registry study. *BMC Nephrol.* 16:22. 10.1186/s12882-015-0002-z 25886171 PMC4359504

[B15] Esteva A. Kuprel B. Novoa R. A. Ko J. Swetter S. M. Blau H. M. (2017). Dermatologist-level classification of skin cancer with deep neural networks. *Nature* 542 115–118. 10.1038/nature21056 28117445 PMC8382232

[B16] Fessenden T. B. Beckham Y. Perez-Neut M. Ramirez-San Juan G. Chourasia A. H. Macleod K. F. (2018). Dia1-dependent adhesions are required by epithelial tissues to initiate invasion. *J. Cell Biol.* 2174 1485–1502. 10.1083/jcb.201703145 29437785 PMC5881494

[B17] Freelon D. G. (2010). ReCal: intercoder reliability calculation as a web service. *Int. J. Internet Sci.* 51 20–33.

[B18] Freelon D. G. (2013). ReCal OIR: ordinal, interval, and ratio intercoder reliability as a web service. *Int. J. Internet Sci.* 81 10–16.

[B19] Füllekrug J. Shevchenko A. Shevchenko A. Simons K. (2006). Identification of glycosylated marker proteins of epithelial polarity in MDCK cells by homology driven proteomics. *BMC Biochem.* 7:8. 10.1186/1471-2091-7-8 16533391 PMC1421407

[B20] Galvez-Santisteban M. Rodriguez-Fraticelli A. E. Bryant D. M. Vergarajauregui S. Yasuda T. Banon-Rodriguez I. (2012). Synaptotagmin-like proteins control the formation of a single apical membrane domain in epithelial cells. *Nat. Cell Biol.* 148 838–849. 10.1038/ncb2541 22820376 PMC3433678

[B21] Giles R. H. Ajzenberg H. Jackson P. K. (2014). 3D spheroid model of mIMCD3 cells for studying ciliopathies and renal epithelial disorders. *Nat. Protoc.* 912 2725–2731. 10.1038/nprot.2014.181 25356583

[B22] Gupta A. Harrison P. J. Wieslander H. Pielawski N. Kartasalo K. Partel G. (2019). Deep learning in image cytometry: a review. *Cytometry A* 954 366–380. 10.1002/cyto.a.23701 30565841 PMC6590257

[B23] Hartnett M. E. (2010). “Breakdown of the RPE blood–retinal barrier,” in *The Retina and its Disorders*, eds Besharse J. C. Bok D. (Oxford: Academic Press), 58–67.

[B24] Hildebrandt F. Benzing T. Katsanis N. (2011). Ciliopathies. *N. Engl. J. Med.* 36416 1533–1543. 10.1056/NEJMra1010172 PMC3640822 21506742

[B25] Huang X. (2013). *Cell2string, MATLAB Central File Exchange*. Available online at: http://de.mathworks.com/matlabcentral/fileexchange/39532-cell2string (accessed Februrary 24, 2017).

[B26] Hynes A. M. Giles R. H. Srivastava S. Eley L. Whitehead J. Danilenko M. (2014). Murine Joubert syndrome reveals Hedgehog signaling defects as a potential therapeutic target for nephronophthisis. *Proc. Natl. Acad. Sci. U.S.A.* 11127 9893–9898. 10.1073/pnas.1322373111 24946806 PMC4103340

[B27] Ivers L. P. Cummings B. Owolabi F. Welzel K. Klinger R. Saitoh S. (2014). Dynamic and influential interaction of cancer cells with normal epithelial cells in 3D culture. *Cancer Cell Int.* 141:108. 10.1186/s12935-014-0108-6 PMC4221723 25379014

[B28] Kermany D. S. Goldbaum M. Cai W. Valentim C. C. S. Liang H. Baxter S. L. (2018). Identifying medical diagnoses and treatable diseases by image-based deep learning. *Cell* 1725 1122.e9–1131.e9. 10.1016/j.cell.2018.02.010 29474911

[B29] Khan A. Sohail A. Zahoora U. (2019). A survey of the recent architectures of deep convolutional neural networks. *arXiv* [preprint] Available online at: https://arxiv.org/ftp/arxiv/papers/1901/1901.06032.pdf (accessed February 14, 2020).

[B30] Kovacic J. C. Mercader N. Torres M. Boehm M. Fuster V. (2012). Epithelial-to-mesenchymal and endothelial-to-mesenchymal transition. *Circulation* 125 1795–1808. 22492947 10.1161/CIRCULATIONAHA.111.040352 PMC3333843

[B31] Le N. Ho Q. Ou Y. (2018). Classifying the molecular functions of Rab GTPases in membrane trafficking using deep convolutional neural networks. *Anal. Biochem.* 555 33–41. 10.1016/j.ab.2018.06.011 29908156

[B32] Le N. Q. K. Huynh T. Yapp E. K. Y. Yeh H. (2019). Identification of clathrin proteins by incorporating hyperparameter optimization in deep learning and PSSM profiles. *Comput. Methods Programs Biomed.* 177 81–88. 10.1016/j.cmpb.2019.05.016 31319963

[B33] LeCun Y. Bengio Y. Hinton G. (2015). Deep learning. *Nature* 521 436–444. 26017442 10.1038/nature14539

[B34] Martin-Belmonte F. Yu W. Rodriguez-Fraticelli A. E. Ewald A. J. Werb Z. Alonso M. A. (2008). Cell-polarity dynamics controls the mechanism of lumen formation in epithelial morphogenesis. *Curr. Biol.* 187 507–513. 10.1016/j.cub.2008.02.076 18394894 PMC2405957

[B35] McHugh M. L. (2012). Interrater reliability: the kappa statistic. *Biochem. Med.* 223 276–282. 10.11613/bm.2012.031 PMC3900052 23092060

[B36] Meder D. Shevchenko A. Simons K. Fullekrug J. (2005). Gp135/podocalyxin and NHERF-2 participate in the formation of a preapical domain during polarization of MDCK cells. *J. Cell Biol.* 1682 303–313. 10.1083/jcb.200407072 15642748 PMC2171597

[B37] Moen E. Bannon D. Kudo T. Graf W. Covert M. Van Valen D. (2019). Deep learning for cellular image analysis. *Nat. Methods* 16 1233–1246. 31133758 10.1038/s41592-019-0403-1 PMC8759575

[B38] Monjaret F. Fernandes M. Duchemin-Pelletier E. Argento A. Degot S. Young J. (2016). Fully automated one-step production of functional 3D tumor spheroids for high-content screening. *J. Lab. Autom.* 212 268–280. 10.1177/2211068215607058 26385905

[B39] O’Brien L. E. Zegers M. M. Mostov K. E. (2002). Opinion: building epithelial architecture: insights from three-dimensional culture models. *Nat. Rev. Mol. Cell Biol.* 37 531–537. 10.1038/nrm859 12094219

[B40] Okuyama H. Kondo J. Sato Y. Endo H. Nakajima A. Piulats J. M. (2016). Dynamic change of polarity in primary cultured spheroids of human colorectal adenocarcinoma and its role in metastasis. *Am. J. Pathol.* 1864 899–911. 10.1016/j.ajpath.2015.12.011 26878211

[B41] Oshima T. Miwa H. (2016). Gastrointestinal mucosal barrier function and diseases. *J. Gastroenterol.* 518 768–778. 10.1007/s00535-016-1207-z 27048502

[B42] Petridou N. I. Skourides P. A. (2014). FAK transduces extracellular forces that orient the mitotic spindle and control tissue morphogenesis. *Nat. Commun.* 5:5240. 10.1038/ncomms6240 25341507

[B43] Ravi M. Paramesh V. Kaviya S. R. Anuradha E. Solomon F. D. (2015). 3D cell culture systems: advantages and applications. *J. Cell. Physiol.* 2301 16–26. 10.1002/jcp.24683 24912145

[B44] Rodriguez-Fraticelli A. E. Auzan M. Alonso M. A. Bornens M. Martin-Belmonte F. (2012). Cell confinement controls centrosome positioning and lumen initiation during epithelial morphogenesis. *J. Cell Biol.* 1986 1011–1023. 10.1083/jcb.201203075 22965908 PMC3444774

[B45] Rodriguez-Fraticelli A. E. Martin-Belmonte F. (2013). Mechanical control of epithelial lumen formation. *Small GTPases* 42 136–140. 10.4161/sgtp.24303 23511851 PMC3747256

[B46] Roignot J. Peng X. Mostov K. (2013). Polarity in mammalian epithelial morphogenesis. *Cold Spring Harb. Perspect. Biol.* 52:a013789. 10.1101/cshperspect.a013789 23378592 PMC3552506

[B47] Scheeder C. Heigwer F. Boutros M. (2018). Machine learning and image-based profiling in drug discovery. *Curr. Opin. Syst. Biol.* 10 43–52. 10.1016/j.coisb.2018.05.004 30159406 PMC6109111

[B48] Schindelin J. Arganda-Carreras I. Frise E. Kaynig V. Longair M. Pietzsch T. (2012). Fiji: an open-source platform for biological-image analysis. *Nat. Methods* 97 676–682. 10.1038/nmeth.2019 22743772 PMC3855844

[B49] Schindelin J. Rueden C. T. Hiner M. C. Eliceiri K. W. (2015). The ImageJ ecosystem: an open platform for biomedical image analysis. *Mol. Reprod. Dev.* 827 518–529. 10.1002/mrd.22489 26153368 PMC5428984

[B50] Shin H. Roth H. R. Gao M. Lu L. Xu Z. Nogues I. (2016). Deep convolutional neural networks for computer-aided detection: CNN architectures, dataset characteristics and transfer learning. *IEEE Trans. Med. Imaging* 355 1285–1298. 10.1109/tmi.2016.2528162 26886976 PMC4890616

[B51] Slaats G. G. Lilien M. R. Giles R. H. (2016). Nephronophthisis: should we target cysts or fibrosis? *Pediatr. Nephrol.* 314 545–554. 10.1007/s00467-015-3162-y 26219413

[B52] Sommer C. Gerlich D. W. (2013). Machine learning in cell biology - teaching computers to recognize phenotypes. *J. Cell. Sci.* 12624:5529. 10.1242/jcs.123604 24259662

[B53] Szegedy C. Liu W. Jia Y. Sermanet P. Reed S. Anguelov D. (2015). “Going deeper with convolutions,” *2015 IEEE Conference on Computer Vision and Pattern Recognition (CVPR)* (Boston, MA: IEEE), 1–9. 10.1109/CVPR.2015.7298594

[B54] Tang B. Pan Z. Yin K. Khateeb A. (2019). Recent advances of deep learning in bioinformatics and computational biology. *Front. Genet.* 10:214 10.3389/fgene.2019.00214 PMC6443823 30972100

[B55] Tsochatzidis L. Costaridou L. Pratikakis I. (2019). Deep learning for breast cancer diagnosis from mammograms: a comparative study. *J. Imaging* 5:37 10.3390/jimaging5030037 PMC8320909 34460465

[B56] Wang Q. Margolis B. (2007). Apical junctional complexes and cell polarity. *Kidney Int.* 7212 1448–1458. 10.1038/sj.ki.5002579 17914350

[B57] Ware S. M. Aygun M. G. Hildebrandt F. (2011). Spectrum of clinical diseases caused by disorders of primary cilia. *Proc. Am. Thorac. Soc.* 85 444–450. 10.1513/pats.201103-025sd 21926397 PMC3209578

[B58] Waters A. M. Beales P. L. (2011). Ciliopathies: an expanding disease spectrum. *Pediatr. Nephrol.* 267 1039–1056. 10.1007/s00467-010-1731-7 21210154 PMC3098370

[B59] Weigert M. Schmidt U. Boothe T. Müller A. Dibrov A. Jain A. (2018). Content-aware image restoration: pushing the limits of fluorescence microscopy. *Nat. Methods* 1512 1090–1097. 10.1038/s41592-018-0216-7 30478326

[B60] Yao K. Rochman N. D. Sun S. X. (2019). Cell type classification and unsupervised morphological phenotyping from low-resolution images using deep learning. *Sci. Rep.* 91:13467. 10.1038/s41598-019-50010-9 PMC6749053 31530889

[B61] Yonemura S. (2014). Differential sensitivity of epithelial cells to extracellular matrix in polarity establishment. *PLoS One* 911:e112922. 10.1371/journal.pone.0112922 25393292 PMC4231087

