# Supplementary material for: Application and Comparison of Supervised Learning Strategies to Classify Polarity of Epithelial Cell Spheroids in 3D Culture
Source: Front Genet. 2020 Mar 27;11:248. doi: 10.3389/fgene.2020.00248 (PMC7119422; doi:10.3389/fgene.2020.00248)
Supplement: Supplementary file 1 [file Data_Sheet_1.PDF]

# *Supplementary Material*

## 1 Supplementary Material and Methods

*Experimental conditions of spheroid assay*

MDCK II cells were transfected with siRNA (Table S1) according to manufacturer's protocol using amaxa cell line nucleofection kit L (Lonza, Basel, Switzerland). 48h past transfection single cells were seeded on kollagen-coated disc-shaped micropattern of 700 µm². MEM containing 2% FBS, 200mM L-glutamine and 1% penicillin/streptomycin was used. After 4 hours, half of the medium is replaced by MEM containing in addition 5% matrigel (Matrigel Basement Membrane Matrix; Corning, New York, USA). Blebbistatin treatment (40µM, B0560, Sigma-Aldrich, Darmstadt, Germany) was performed eighter on day 1, 2 or 3. After 24 h media was replaced stepwise by washing 3x with MEM containing in addition 2,5% matrigel.

## 2 Supplementary Figures and Tables

**Table S1: siRNA sequences**

| | sense (5'→3') | antisense (5'→3') |
|---|---|---|
| siPkhd1 | AAGCAUCAAAUCCGAGUCCGU | ACGGACUCGGAUUUGAUGCUU |
| siControl | CGUACGCGGAAUACUUCGATT | AAUCGAAGUAUUCCGCGUACG |

### 2.1 Supplementary Figures

**Figure S1**

**Scheme of image processing by ImageJ/FIJI macros for machine learning-based classifier.** (A) *4- colour fluorescence images*; The analysis is based on epithelial cell spheroids stained for basolateral (green) and apical (red) markers, F-actin (magenta) and nuclei (blue). For each spheroid, a z-stack of 50 image planes is used. (B) *Equatorial plane projection*; The function <Midplane> extracts seven equatorial planes, performs an average intensity projection followed by subtraction of the background, and saves the resulting image file for further use in MATLAB. (C) *Shape parameters of spheroid*; The function <MaxRadius> performs a grouped summed intensity projection of all planes and all channels. A binary mask of the whole spheroid is generated and shape parameters are determined. By drawing the boundary of the spheroid and measuring the distance to the centre of mass, the <maximum radius> is calculated. (D) *Shape parameters of the actin network*; The function <Actinbelt> analyses the actin signal based on an average intensity projection and a threshold of the peak signal (6 %). The resulting actin particles provide luminal size and count. (E) *Shape parameters of nuclear positions*; The function <CentreNuclei> determines the centre of mass and other parameters of the nuclei using integrated intensity projections and generation of a binary mask.

**Figure S2**

**Scheme of operations within MATLAB providing parameters for machine learning.** A 4-colour fluorescence image of the spheroid's equatorial plane is converted to a cumulative intensity plot for each channel, relative to the centre of mass (CoM). (A) *Polar transformation*; Visualisation of the angle independent polar transformation P(x,y) to P(ϕ,r), which reduces x,y-coordinates to a distance from the CoM. (B) *Radial distribution of markers*; Plot of cumulative intensities normalized to total intensity of each marker channel (i.e. colour) versus radial distance normalized to the maximum radius of the spheroid. Dashed lines indicate positions used for signal segregation and interpretation, (i) radius at 60% of cumulative signal intensity (r_channel) and (ii) nuclear intensity at 30 % of spheroid radius (y_nuclei). (C) *List of numerical parameters;* Features describing the shape of spheroids and position of fluorescence signals.

**Figure S3**

**Classifier training – Complex Decision Tree for spheroid classification.**

*Example of Complex Decision Tree*; Using the classification learner app of the MATLAB "Statistics and Machine Learning Toolbox" a Complex Decision Tree was trained based on a subset of already classified spheroids (n>20 each group), using variation of the predictor subset and 20-fold cross validation. This process allows adaptation of spheroid classification to variations in assay conditions. Abbreviations: r_channel: radius at 60% of cumulative signal intensity; Δ r_gp58-r_gp135: difference r_gp58 to r_gp135; Δ CoM nuclei-spheroid: difference centres of mass nuclei to spheroid.

**Figure S4**

**Workflow of retraining CNN networks.** In pretrained networks, which are optimally trained on similar data, classification specific final layers can be identified and replaced with empty layers. Subsequent training of the network with labelled training data leads to new specific classification layers, connecting information from all previous layers to image labels or here, polarity groups. Network training comprises internal validation step, wherein parts of the training data are used to control accuracy of prediction.

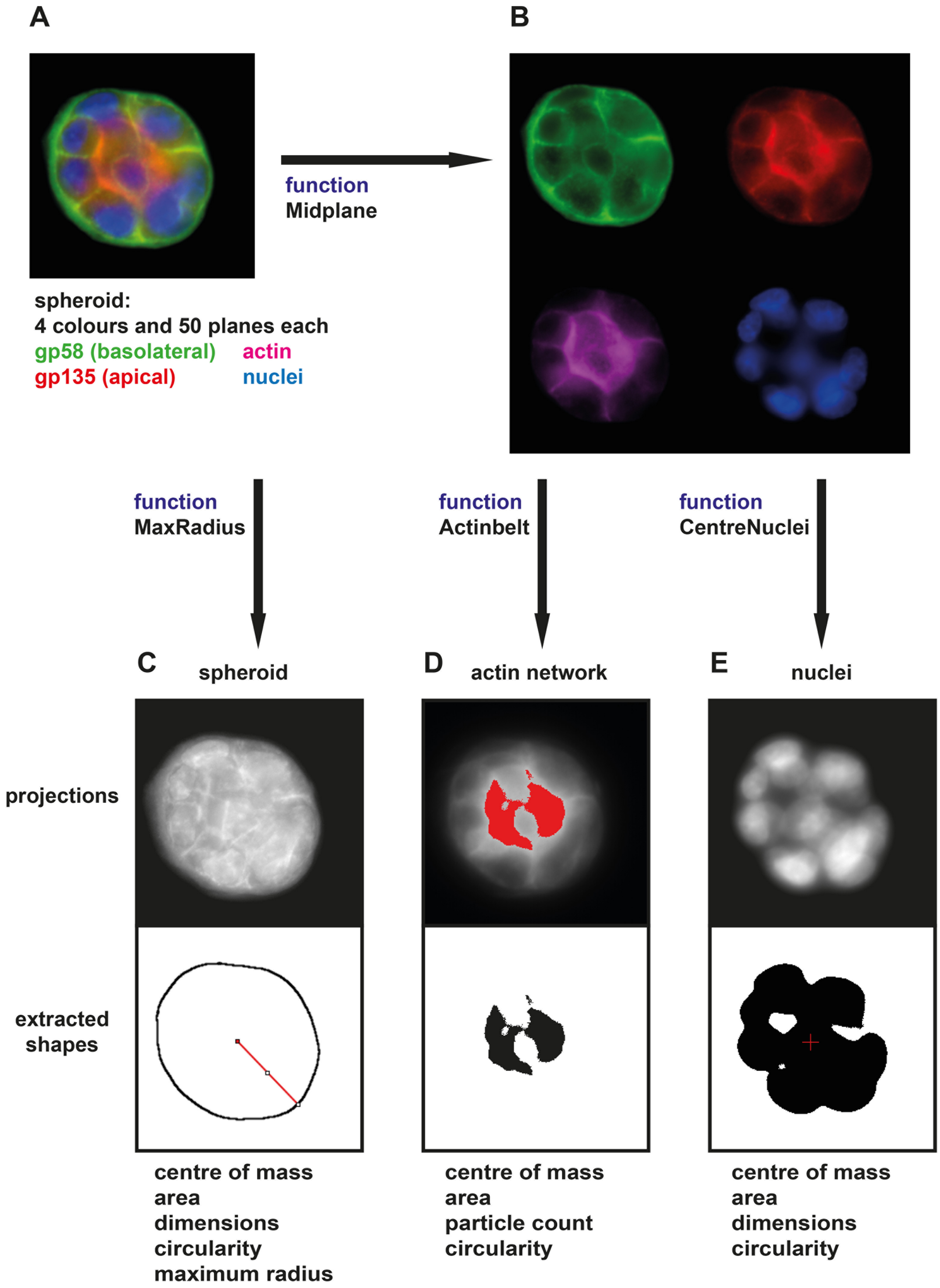
A
B
function
Midplane
spheroid:
4 colours and 50 planes each
gp58 (basolateral)
actin
gp135 (apical)
nuclei
function
MaxRadius
function
Actinbelt
function
CentreNuclei
C
spheroid
D
actin network
E
nuclei
projections
extracted shapes
centre of mass
area
dimensions
circularity
maximum radius
centre of mass
area
particle count
circularity
centre of mass
area
dimensions
circularity

Figure S1

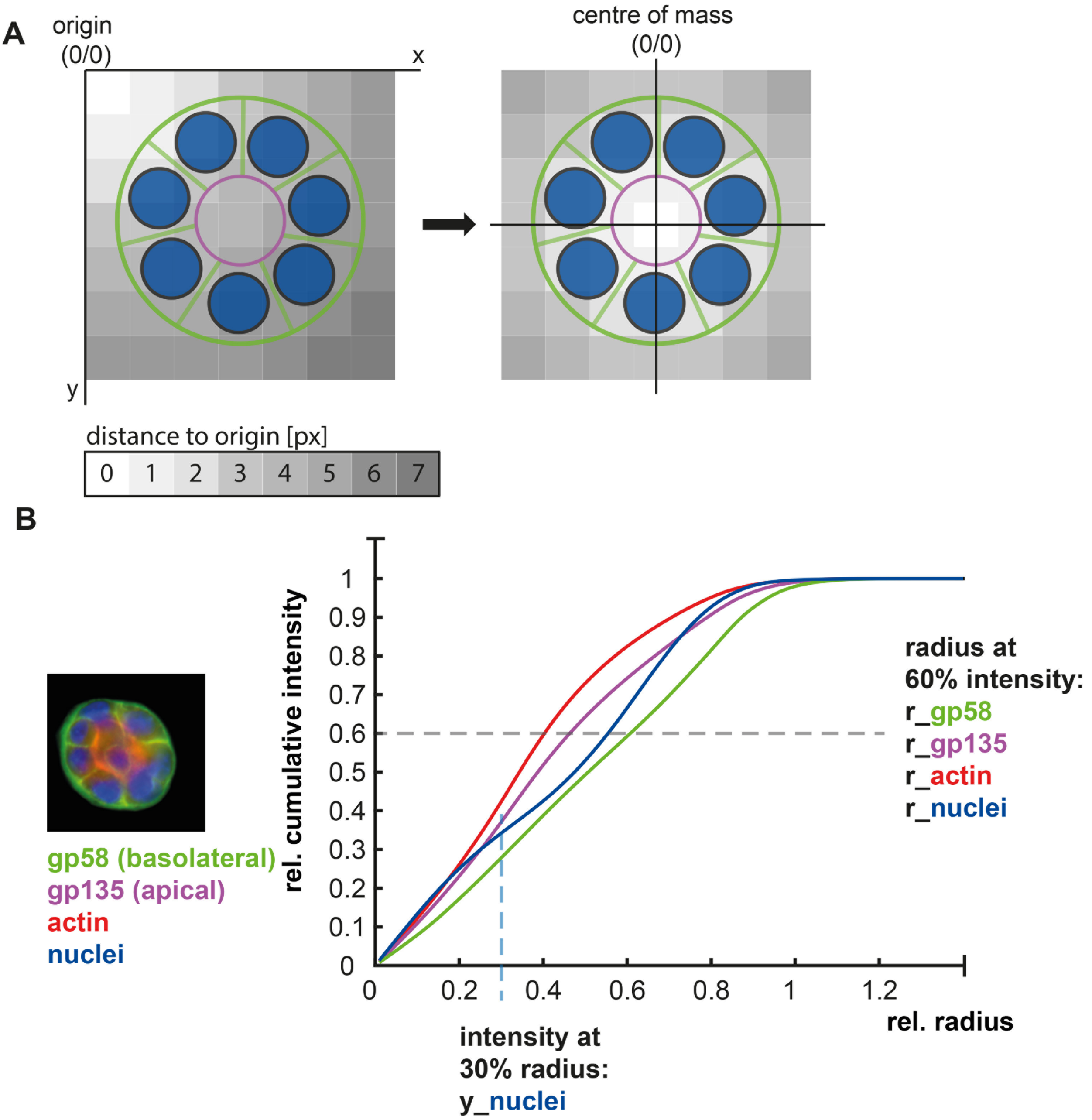


**C**

(1) difference of polarity markers (gp58-gp135)
(2) difference of actin-gp135
(3) r_gp58
(4) r_gp135
(5) r_actin
(6) r_nuclei
(7) circularity of spheroid
(8) circularity of nuclei
(9) average initial slope of nuclear signal
(10) amount of actin area
(11) actin particle count
(12) actin particle count after watershed
(13) circularity of actin
(14) distance CoM nuclei-spheroid
(15) distance CoM nuclei-actin

Figure S2

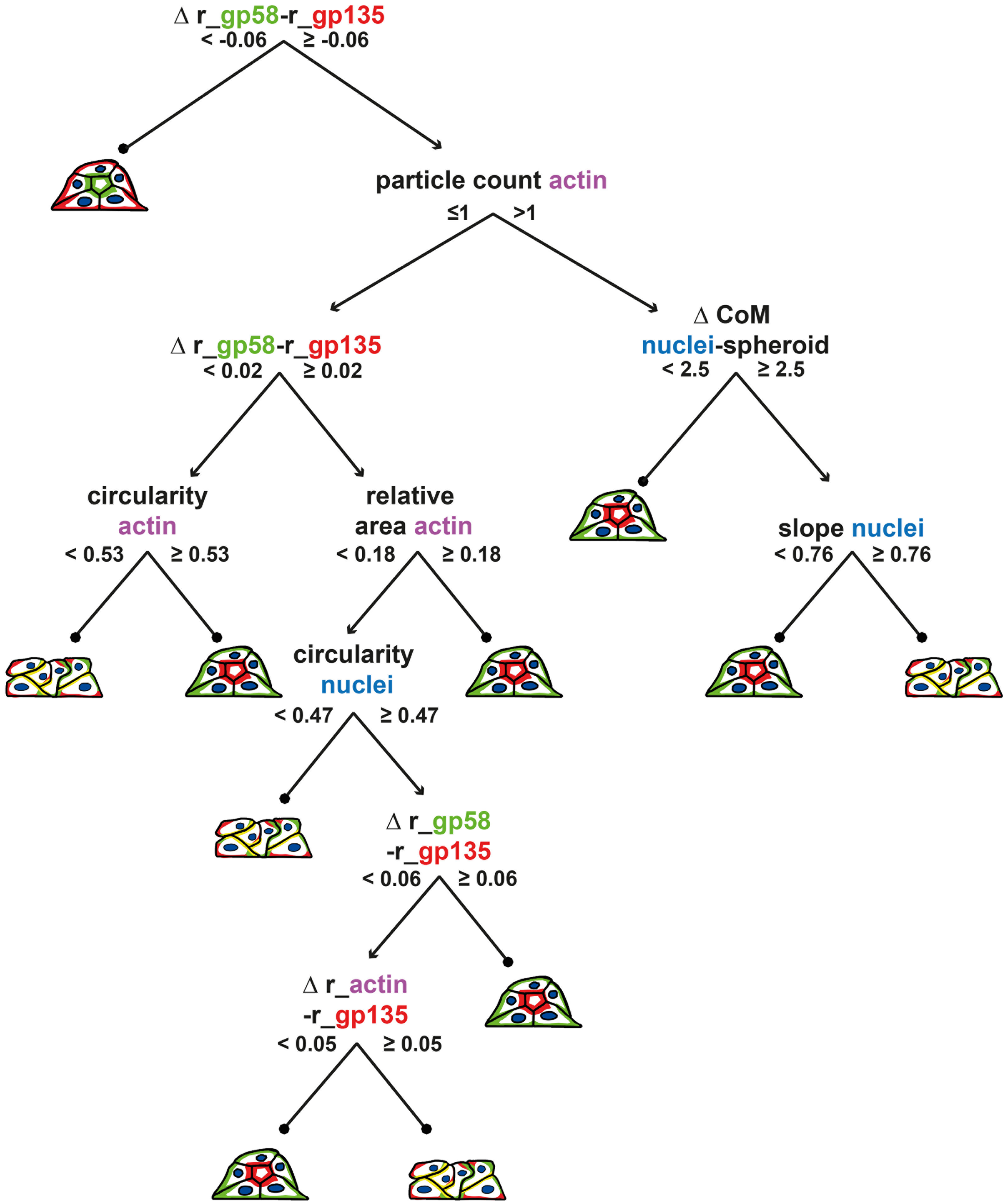
Δ r_gp58-r_gp135
< -0.06
≥ -0.06
particle count actin
≤1
>1
Δ r_gp58-r_gp135
< 0.02
≥ 0.02
Δ CoM
nuclei-spheroid
< 2.5
≥ 2.5
circularity
actin
< 0.53
≥ 0.53
relative
area actin
< 0.18
≥ 0.18
slope nuclei
< 0.76
≥ 0.76
circularity
nuclei
< 0.47
≥ 0.47
Δ r_gp58
-r_gp135
< 0.06
≥ 0.06
Δ r_actin
-r_gp135
< 0.05
≥ 0.05

Figure S3

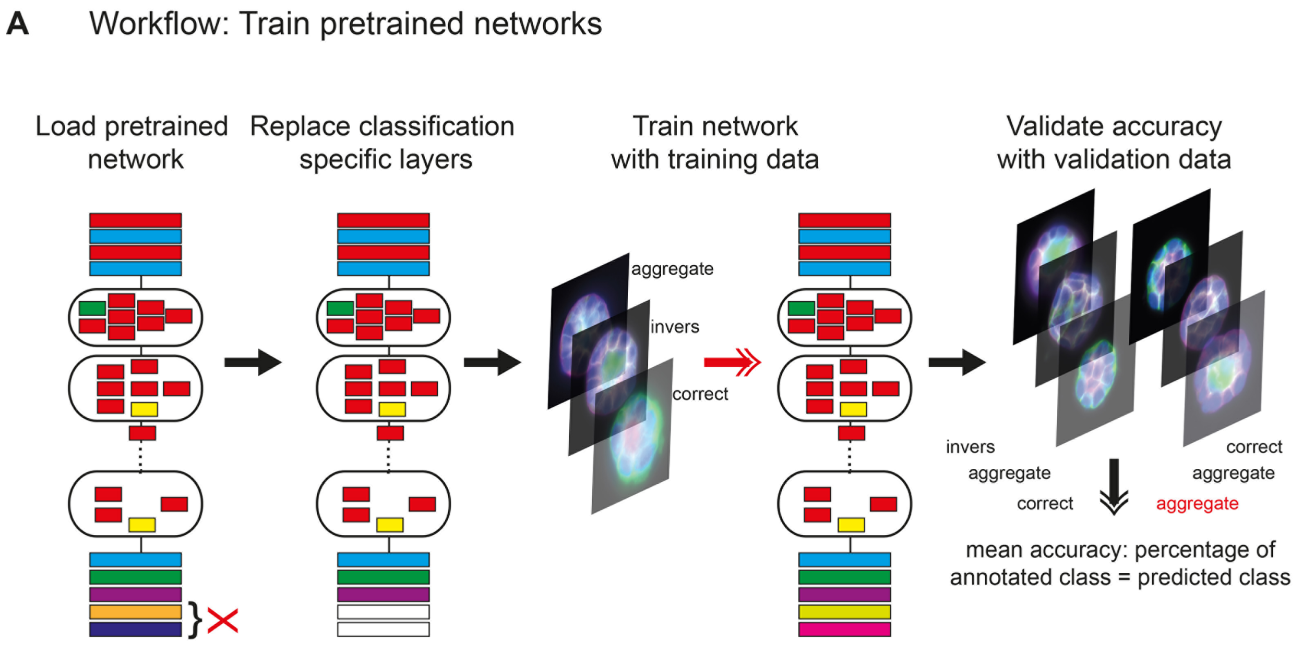
A
Workflow: Train pretrained networks
Load pretrained network
Replace classification specific layers
Train network with training data
Validate accuracy with validation data
aggregate
invers
correct
invers
aggregate
correct
correct
aggregate
aggregate
mean accuracy: percentage of annotated class = predicted class

Figure S4
